# Differentiating percussion pits and carnivore tooth pits using 3D reconstructions and geometric morphometrics

**DOI:** 10.1371/journal.pone.0194324

**Published:** 2018-03-28

**Authors:** José Yravedra, Julia Aramendi, Miguel Ángel Maté-González, Lloyd Austin Courtenay, Diego González-Aguilera

**Affiliations:** 1 Department of Prehistory, Complutense University, Profesor Aranguren s/n, Madrid, Spain; 2 IDEA (Institute of Evolution in Africa), Origins Museum, Plaza de San Andrés 2, Madrid, Spain; 3 Department of Cartographic and Land Engineering, Higher Polytechnic School of Avila, University of Salamanca, Hornos Caleros 50, Avila, Spain; 4 C.A.I. Arqueometry and Archaeological Analysis, Complutense University, Profesor Aranguren s/n, Madrid, Spain; Institut Català de Paleoecologia Humana i Evolució Social (IPHES), SPAIN

## Abstract

During the end of the 20th century and the beginning of the 21st century the discussion on early human behavioral patterns revolved around the *hunting versus scavenging* debate. The correct identification of bone modifications, including percussion, cut and tooth marks, is a key issue within this debate. While many authors have shown that carnivore and human modifications can be easily distinguished, it is true that sometimes percussion marks without associated microstriations and tooth pits overlap morphologically, causing confusion, especially when unmodified hammerstones are used. In order to solve this equifinality problem, many investigations have focused their efforts on other pieces of evidence such as the identification of notches, fragmentation patterns and frequencies, among others. These studies, however, cannot be considered as fully conclusive. Within this paper we address the problem of equifinality when identifying percussion marks produced with unmodified hammerstones and tooth pits created by carnivores using new methodologies based on the 3D reconstruction of marks and their statistical multivariate analysis. For the purpose of this study a total of 128 marks– 39 percussion marks produced with an unmodified quartzite hammerstone, and 89 pits generated by different carnivores–were virtually modelled with the aid of a DAVID structured-light scanner SLS-2 and later analyzed by means of geometric morphometrics. Our results show that percussion marks not associated with striae fields and the pits generated by the carnivores studied here can be successfully distinguished.

## Introduction

Taphonomy is key in the interpretation of archaeological sites. Although the definition of taphonomy was first established by [1], the origins of the discipline may be older [2]. Taphonomy, however, did not actually arise as a scientific discipline within archaeology until 1981. The taphonomic perspective adopted by [3, 4, 5] and other authors, to study the Plio-Pleistocene record marked a new beginning in archaeology. The work conducted by [4] symbolizes the end of a cycle where the australopiths became prey instead of hunters and the osteodontokeratic culture turned into a historiographic myth, whereas [3] proposed a new paradigm explaining the behavior of the first human beings called the man as a scavenger.

Binford's theories on early hominin behavior [3] were discussed and surpassed by a new model, according to which these early populations were specialized scavengers with preference for feline preys that had access to the carcasses before the appearance of hyenas [6–16]. Within this new framework the correct identification of the marks observed on cortical bone became key for the correct interpretation of the Early Paleolithic sites. Not only the presence of certain taphonomic processes, but also the frequency with which they occur became of utmost relevance, since different incidence rates might be indicating human hunting behavior or human scavenging [7–9, 13]. For example, only a few years later it was demonstrated that many of the tooth marks identified at the Early Paleolithic FLK site (Olduvai Gorge, Tanzania) were actually biochemical alterations [17]. This re-analysis suggested that carnivores would not have played such an important role at FLK and that, instead, hominins would have been the main accumulating agent at the site [17–19].

Among bone surface modifications, cut [19, 20] and percussion marks [7, 15], as well as their frequencies, are especially important in the identification of exploitation patterns in the fossil record. The identification of percussion marks can be complicated, though. Percussion marks are, superficially, similar to carnivore tooth pits [8], especially when percussion marks are performed with hammerstones [13]. Given these difficulties, the methods to identify bone surface modifications and the inclusion or exclusion of inconspicuous marks in the analyses have been extensively discussed. Two experimental analyses [21,22] stress the adversities taphonomists must cope with. In [22] percussion marks generated with modified and unmodified hammerstones were analyzed and compared with tooth marks. The study shows that percussion marks produced with modified hammerstones differ from tooth marks, while percussion marks produced with unmodified hammerstones are more problematic, since one third of the marks are not associated with striae fields. That means that 30% of the percussion marks generated with unmodified hammerstones is susceptible to equifinality and can be easily mistaken for tooth marks generated by carnivores, since they cannot be distinguished based on the presence of microstriations or irregular tissue in the surroundings of the percussion mark. In fact, the morphological characterization of percussion marks stated how “*high breadth*: *depth ratio for pits and grooves but internal surface typically lacks crushing*. *Very shallow microstriations in and/or emanating from pits and grooves*, *oriented transverse to the long axis and occurring in dense superficial patches*”, whereas for tooth pits “*high breadth*: *depth ratio*, *with shallow U-shaped cross-section*. *Internal surface shows crushing*. *Microstriations rare*, *occurring in low-density patches*” [13]. These observations clearly notice these differences.

The problems encountered in distinguishing percussion marks and tooth pits in controlled experimental contexts usually increase when analyzing archaeological samples. In these contexts, several factors affecting the cortical bone preservation (e.g. biochemical alterations, abrasion, weathering) can hinder the taphonomic analysis and the correct identification of the marks even more. Determining the accumulating and modifying agent of the Plio-Pleistocene sites has turned out to be quite complicated and has required more than the simple description of marks. Therefore, several alternatives have been investigated to overcome these limitations.

One of the criteria is based on the study of notches. Notches are scars located at the edges of bones, associated with fragmentation processes. Notches are less exposed to taphonomic processes [11] and it is possible to infer the fracturing agent–whether human or carnivore–according to their characteristics. However, there are some problems regarding the process of determining notches because notches on small sized carcasses made by humans and carnivores cannot be fully differentiated [22]. On the contrary, it has been observed that the notches on large-sized carcasses can be successfully classified, especially among cattle bones [11, 22]. Though, when comparing notches on equid and bovid carcasses, the analysis is more complex. Another study [23] concluded that the morphology of notches and the proportions of notch types cannot be differentiated from the results of static loading by carnivores. Thus, the analysis of notches should be used alongside other taphonomic indicators such as tooth, percussion and cut mark frequencies, the distribution of anthropogenic and carnivore bone alterations, fracture angles or fragmentation patterns, etc.

A second approach used to fight the problems concerning the differentiation of human and carnivore agency is based on the study of fracture angles [23–25]. Nevertheless, the methodology proposed [24] in classifying the different fracture angles produced on bovid long bone shafts is not diagnostic when applied to equids [23]. In addition, carnivores generate similar fracture angles to those produced by humans using hammerstones [25].

Further evidence used to differentiate the action of humans and carnivores is the ratio of remaining long bone circumferences alongside the degree of fragmentation observed within the osteological accumulation [26]. Diagenetic alterations and sedimentary pressure present at archaeological sites usually affect the fragmentation degree of the bone samples, increasing the frequency of bone shafts that preserve less than 50% of the shaft circumference and the global fragmentation of the bones. Bone accumulations where most bones preserve 100% of the shaft circumference are very scarce in the archaeological and paleontological record, making it difficult to characterize a fossil accumulation only based on the fragmentation degree and the circumference pattern seen among long bones.

In conclusion, the fragmentation, the degree of shaft circumference, the angles of the fracture planes, the notches and the morphology of the pits / percussion marks are not strictly diagnostic when trying to identify the modifying agent of bone accumulations and are subject to equifinality.

In order to review this issue related to the agent fracturing long bone shafts, we have applied a new methodology based on three-dimensional reconstructions and a geometric morphometric analysis to the study of tooth pits generated by carnivores and percussion marks inflicted with unmodified hammerstones and not associated to microstriae fields. These techniques have already been applied to other taphonomic questions providing promising results e.g. cut marks [27–30] or tooth marks [31, 32]. Our aim with this study is to improve the precision in the identification of these types of marks in order to overcome equifinality [8,13,21,22].

## Methods and samples

### Sample

For the purpose of this study we first analyzed a total of 128 marks, including pits created by different carnivores in a controlled setting and percussion marks produced with unmodified hammerstones. As previously proved [31] the methodology employed in this study is affected by prey carcass so, in order to avoid possible discrepancies due to differences in carcass size, we have limited our sample to 89 pits on adult horse long bones generated by wolves (N 24), hyenas (N 21), jaguars (N 20) and lions (N 24) in captivity at the Cabárceno Nature Park, in Cantabria (Spain) [33, 34].

Along with this experimental sample conducted with carnivores, we analyzed 39 percussion marks experimentally created with an unmodified quartzite hammer. Only percussion marks, that are not associated with microstriations, and can therefore be mistaken for carnivore pits, were selected for the study.

We used unmodified quartzite hammerstones because these are the most widespread tools among Paleolithic sites. Other raw materials such as basalt, chert, quartz, sandstone, etc. can be used as pebbles to strike carcasses. Notwithstanding, all unmodified hammerstones leave the same mark patterns regardless of the raw material due to the uniform surface of the tool. Still, a previous experiment was conducted using quartzite, basalt, and sandstone unmodified hammerstones to prove that point. A total of 56 percussion marks were compared to demonstrate the applicability of the methodology to any kind of unmodified hammer (data in S1 Appendix). A skilled person with expertise in conducting neotaphonomic studies performed both experiments.

### Laser scanner structure-light and virtual reconstruction

**Fig 1:**
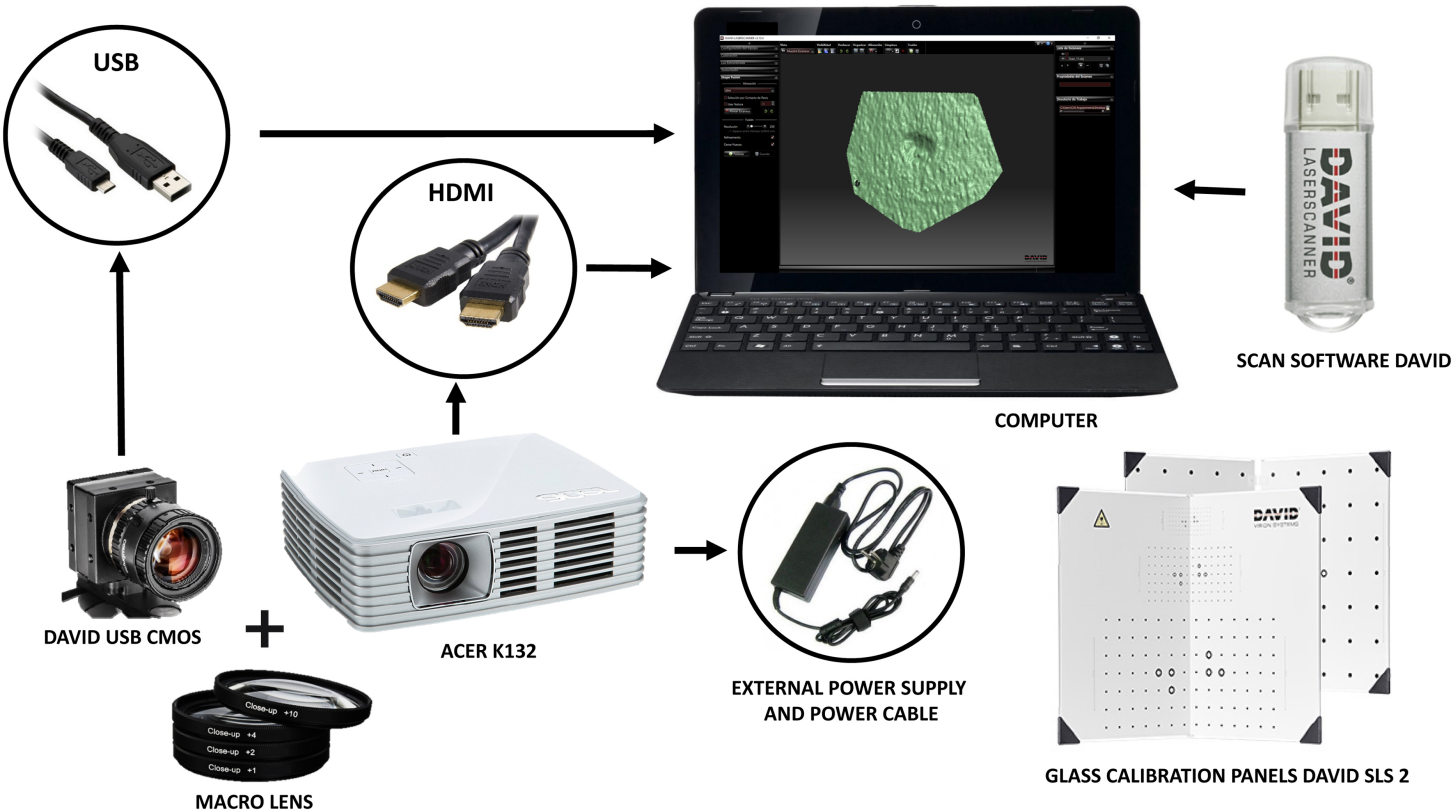
DAVID structured-light scanner SLS-2 technical equipment.

**Fig 2:**
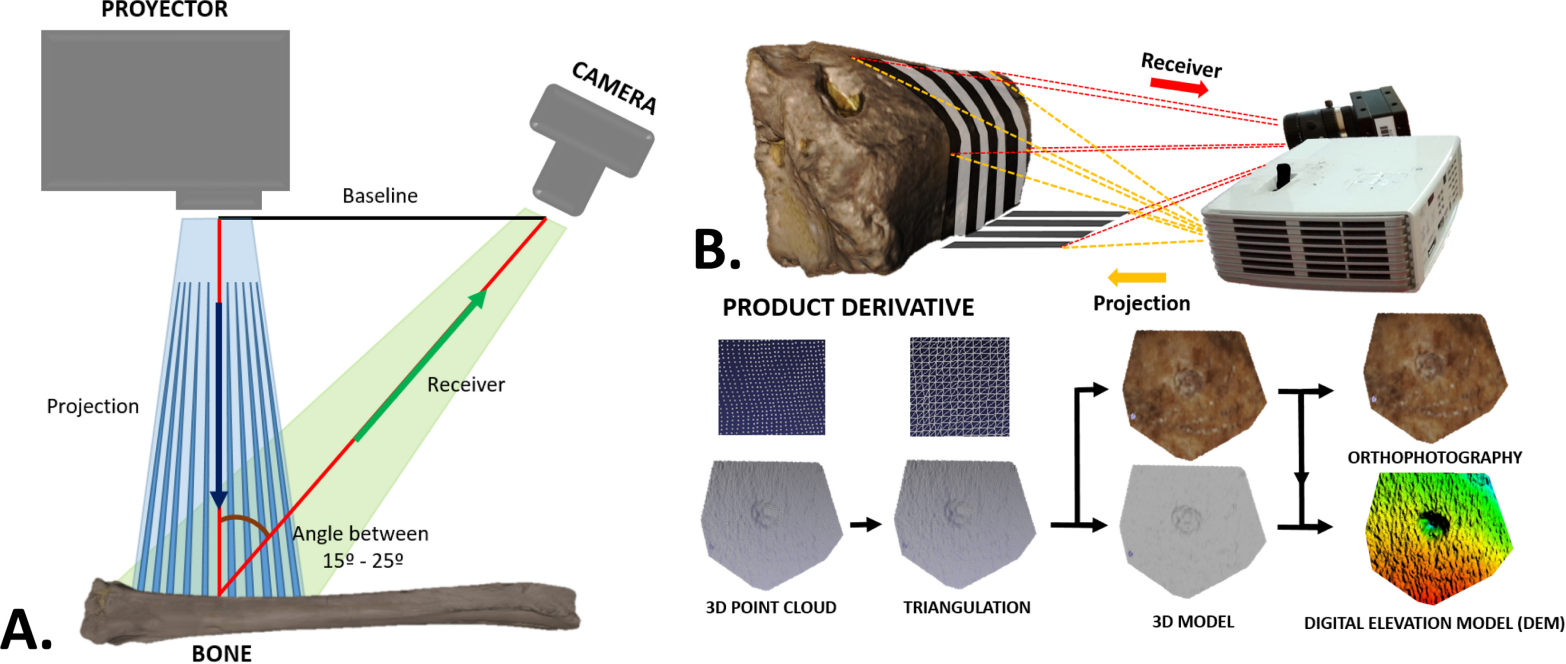
Scanning perform and results. A) Arrangement of the structured-light scanner equipment to perform a 3D scanning. B) 3D scanning and results obtained from data collection.

The process of digitizing the marks was performed with a DAVID structured-light scanner SLS-2 (Table 1) located at the C.A.I. of Archaeometry at the Complutense University of Madrid. The equipment consists of a camera, a projector and a calibration marker board, that in the first phase needs to be calibrated (Fig 1). In order to carry out this process, a DAVID USB CMOS Monochrome camera is positioned and fit with a macro lens alongside an ACER K132 projector, both facing towards the calibration marker board at an angle between 15° and 25° (Fig 2A). The projection produced by the projector has to cover the entire calibration marker board, in our case the size and calibration pattern corresponds to a 15mm scale. Within the DAVID software the scale is introduced as displayed on the calibration marker board, the camera’s exposure is adjusted accordingly while the focus of all the single instruments is adjusted. The equipment is then calibrated. The camera, as well as the projector, have to remain fixed and stable throughout the entire calibration process.

**Table 1:** Technical specifications of the structured light scanner SLS-2.

DAVID structured-light scanner SLS-2	
Workpiece size	16 x 500 mm.
Resolution	Up to 0.1% of scan size (down to ± 0.016 mm).
Scanning time	One single scan within a few seconds.
Mesh density	Up to 1.200.000 vertices per scan.

The second phase consists in substituting the calibration marker board for the bone we intend to scan. The DAVID structured-light scanner SLS-2 is able to produce a density of up to 1.2 million points. The use of this scanning process provides a real reproduction of the bone external topography (Fig 2B). In this case, the matt polished surface of the bones avoids problems related to light intensity, or the contrast of lights and shadows during data collection. The active sensor reduces data capture time to less than 1 minute. The DAVID structured-light scanner SLS-2 [35] used in this experiment produced a higher quality resolution than the scanner used in [27]. This equipment was able to successfully reproduce most of the percussion marks and tooth pits identified on our experimental samples. Inconspicuous marks whose main morphological exterior and interior features could not be appreciated were excluded.

### Geometric morphometrics analysis

**Fig 3:**
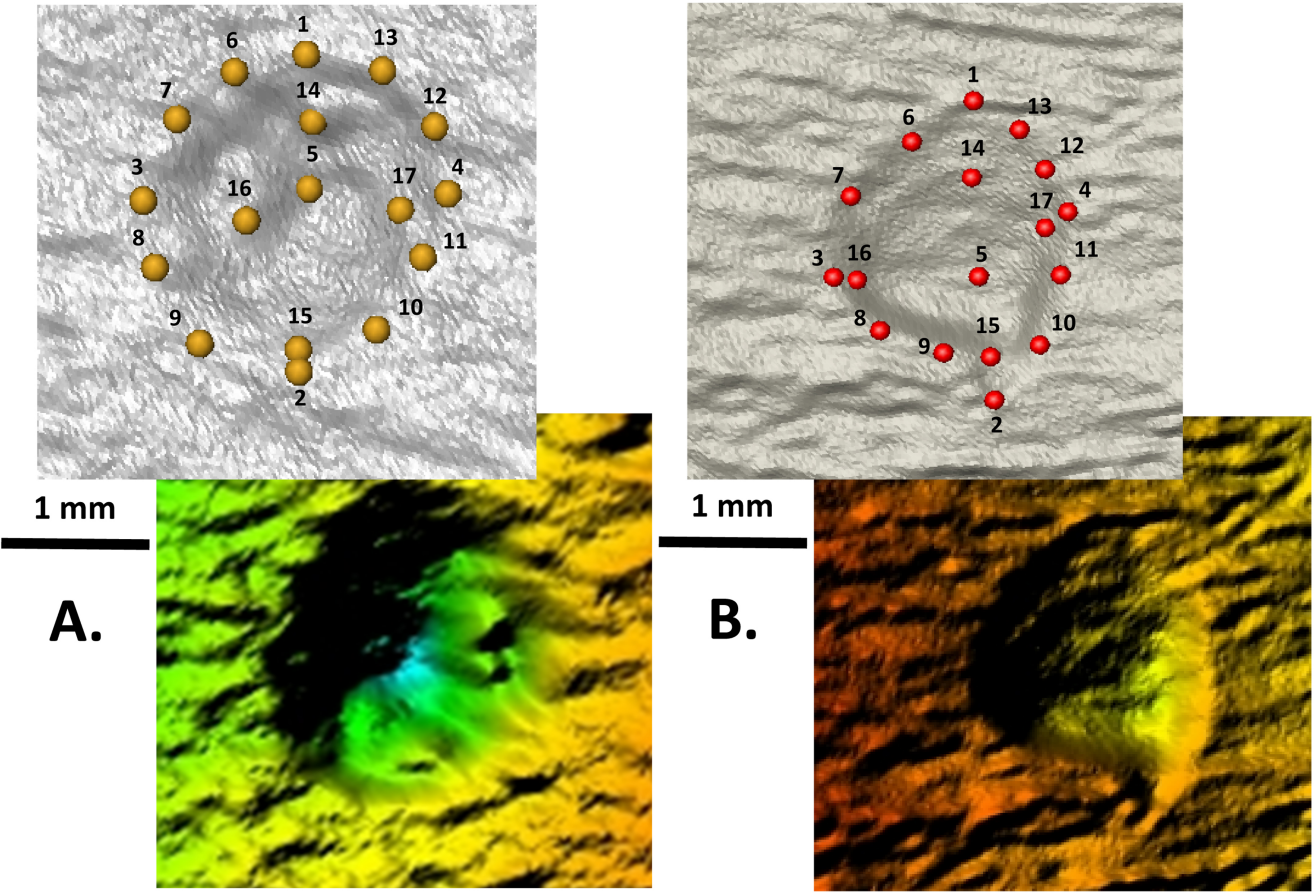
Landmark configurations. A) Percussion marks and B) Tooth pits.

For this analysis, pits and percussion marks were landmarked using 17 three-dimensional points (Table 2) on the exterior and interior surfaces (Fig 3), following [31] methodology. The landmarking step was performed in Avizo (Visualization Sciences Group, USA). A preliminary reliability test was performed to evaluate data collection on percussion marks: two different observers landmarked a preliminary set of the percussion sample (20 marks) demonstrating that there is no significant difference between the data obtained (data in S2 Appendix). The reliability of the landmarking process on tooth pits was already tested in [31].

**Table 2:** List of landmarks used in the study and their description.

N	Landmark	Description
1	Length A	Upper limit of the longitudinal axis
2	Length B	Lower limit of the longitudinal axis
3	Width A	Left limit of the breadth axis
4	Width B	Right limit of the breadth axis
5	Depth	Most centered lowest point of the mark
6	Left upper half A	Point at the first third between the upper limit of the long axis and the left limit of the breadth axis
7	Left upper half B	Point at the second third between the upper limit of the long axis and the left limit of the breadth axis
8	Left lower half A	Point at the first third between the left limit of the breadth axis and the lower limit of the long axis
9	Left lower half B	Point at the second third between the left limit of the breadth axis and the lower limit of the long axis
10	Right upper half A	Point at the first third between the upper limit of the long axis and the right limit of the breadth axis
11	Right upper half B	Point at the second third between the upper limit of the long axis and the right limit of the breadth axis
12	Right lower half A	Point at the first third between the right limit of the breadth axis and the lower limit of the long axis
13	Right lower half B	Point at the second third between the right limit of the breadth axis and the lower limit of the long axis
14	Interior Length A	Upper inflection point on the longitudinal axis
15	Interior Length B	Lower inflection point on the longitudinal axis
16	Interior Width A	Left inflection point on the breadth axis
17	Interior Width B	Right inflection point on the breadth axis

Landmarks contain shape and size information in the form of Cartesian coordinates, allowing the comparison among different elements that can be described in a homologous way [36–39]. Landmark configurations are then analyzed by means of geometric morphometrics based on a Procrustes superimposition, commonly known as generalized procrustes analysis (GPA). This technique takes the landmark data and normalizes the form information by the application of superimposition procedures. This involves the translation, rotation and scaling of shapes defined by landmark configurations. After GPA, there are always some remaining differences that expose patterns of variation and covariation between structures that after being projected into a flat Euclidian space where data can be studied using common multivariate statistics [40–42].

Principal component analyses (PCA) were used to assess patterns of variation among the data in shape and form space to study shape and size differences. PCA is a statistical tool commonly used to reduce large sets of variables to fewer dimensions, simplifying in that way the visualization of the data distribution maintaining the original distances between the specimens. In the PCA plots the data are explained by linear combinations, known as principal components (PCs) that successively account for decreasing proportions of the total sample variance [37]. Form spaces containing size and shape information were obtained by re-scaling data using the natural logarithm of Centroid Size. Centroid size is the measure most commonly used in geometric morphometric studies and is calculated as a composite of the distances between all landmark configurations and their average, the so-called centroid [43]. Changes in shape and form space were visualized with the aid of transformation grids and warpings [44] computed using thin-plate splines in Morphologika 2.5 [45].

Several tests were performed to assess differences and similarities among the sample. The presence of defined groups was statistically tested using a multiple variance analysis (MANOVA) on the PC scores. The test was performed in the free software R [46] to assess differences among the pits generated by different carnivores and the experimentally created percussion marks.

More detailed shape and size analyses were performed to describe morphological changes, since previous analyses on circular marks (e.g. pits) demonstrated that the inclusion of the interior area of such marks causes different results than when only comparing the external morphology of the mark, as it has been traditionally done [31]. Partial least square (PLS) analyses permit the evaluation of two different sets of landmarks, without assuming that one block is dependent on the other [47]. In this case, PLS analyses were conducted to assess the association among the interior and exterior morphology of the marks. Within configuration PLS analyses were conducted to fully address the covariation between the interior and exterior morphology of the pits in the context of the structure, including changes related to the relative sizes, positions and orientations of the data. Two-block PLS analyses were also performed to evaluate shape and size separately [47]. PLS results obtained for the percussion marks were compared with the PLS results obtained for carnivore pits in a previous work [31].

Canonical variate analyses (CVA) and linear discriminant analyses (LDA) were performed to determine the shape features that best distinguish between carnivore pits and percussion marks [48]. CVA and LDA provide differences among groups in Procrustes–the square root of summed squared landmark distances from their centroid–and Mahalanobis distances–distance between points scaled by the within-group variance and correlation [49]. A priori defined groups of carnivore pits and percussion marks were first tested, and a second sample division was created by sub-dividing the carnivore sample into pits generated by lions, hyenas, jaguars and wolves. LDA comparisons were performed in pairs and permutation tests were computed to assess differences between group means.

First, the sample was analyzed including all the marks, and secondly excluding the tooth pits generated by wolves. This carnivore was excluded in a second round because we wanted to generate a sub-sample including only felids and hyaenids that could be applied to the African continent. The generation of this sub-sample could be of utmost importance in deciphering the processes in which different carnivore agents were involved during the early stages of human evolution.

## Results

**Fig 4:**
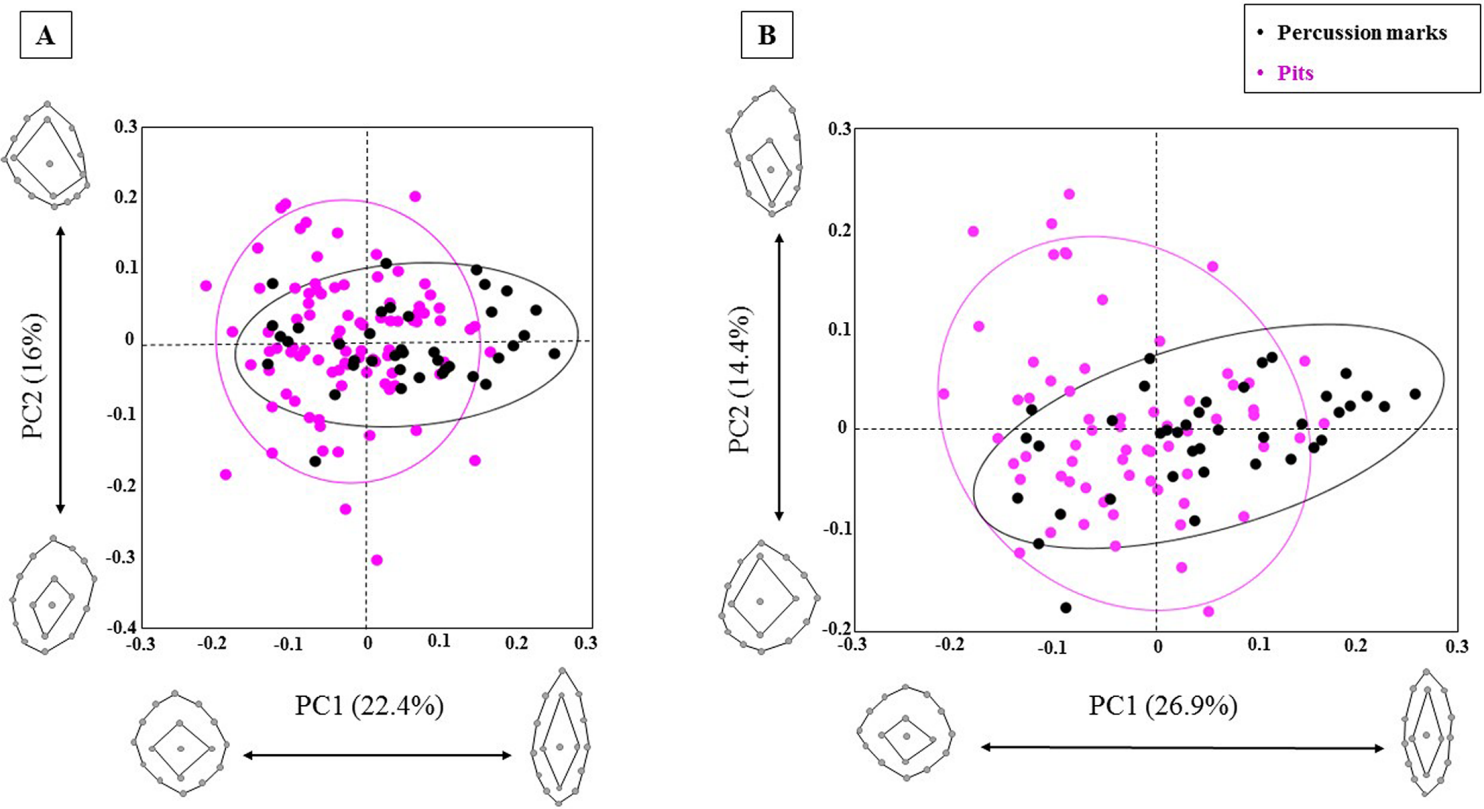
PCA plots in shape space. A) including the whole carnivore pit and percussion sample, B) exluding wolves. Shape changes are visualized for PC1 and PC2 positive and negative axis ends.

**Fig 5:**
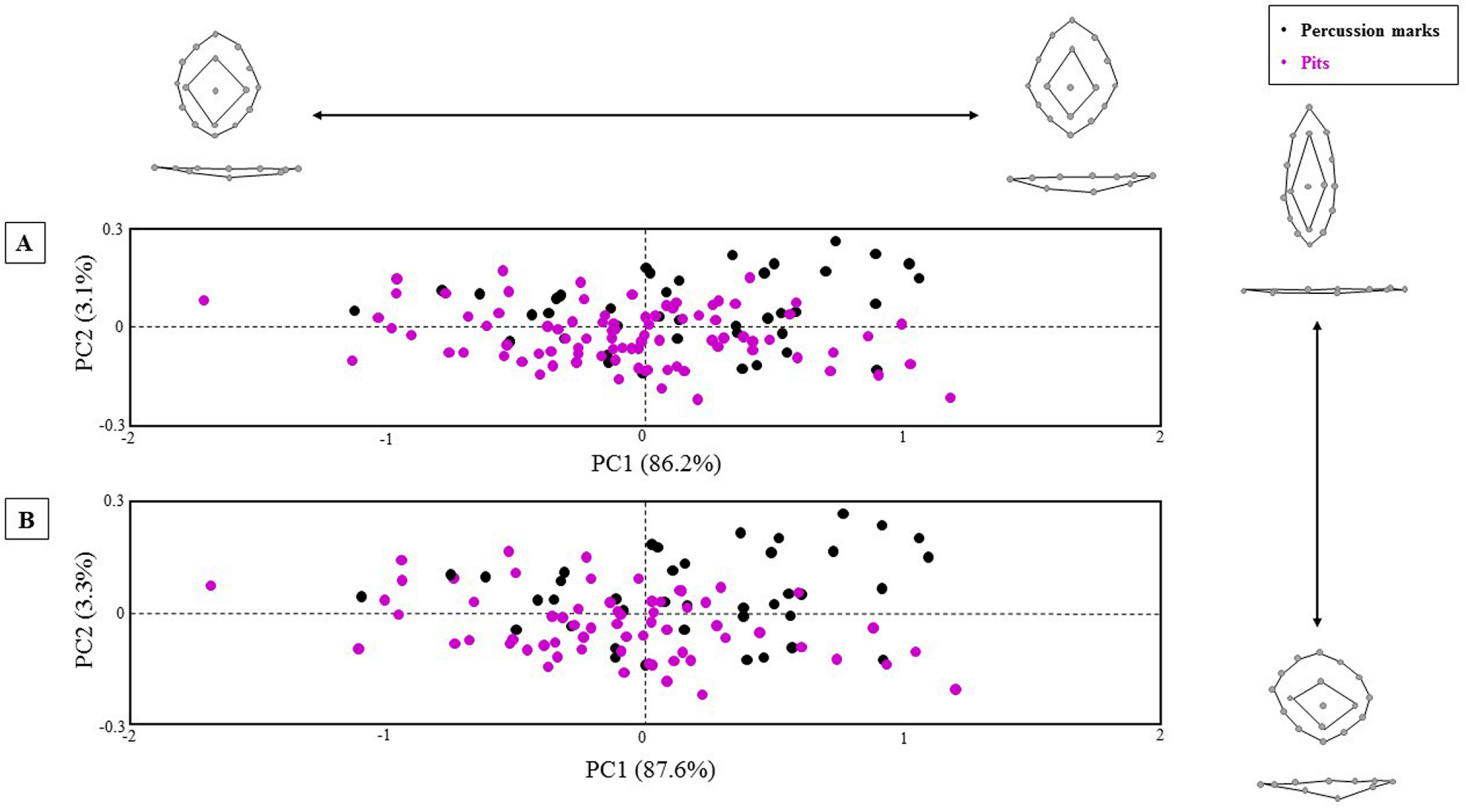
PCA plots in form space. A) including the whole carnivore pit and percussion sample, B) exluding wolves. Form changes are visualized for PC1 and PC2 positive and negative axis ends.

The PCAs of the carnivore pits and percussion marks in shape (Fig 4) and form space (Fig 5) show an important overlapping degree. However, some patterns can be recognized according to mark type.

In the shape space scatter-plot (Fig 4), the first PC appears to be mostly related to changes in width and length of the overall shape on both PCA graphs. Percussion marks tend to follow the trend described by PC1, with most percussion marks closer to more elongated shapes along the positive x-axis. The second PC in shape space is related to the expansion of the interior area. Shape changes expressed by PC2 vary slightly when wolves are not plotted against the graph, and differences in length and width play a greater role. In both PCAs changes along PC2 mainly express shape variance among carnivore pits. These graphs were also observed taking a closer look at the dispersion of individual species. When pits were plotted against the graph using different colors for each carnivore, we noticed that hyena and jaguar pits tend to vary similarly in both directions, while lions and wolves change much more starkly along PC2. In PC1 as well as in PC2 changes in the depth of the pits are also relevant. PC3 (13.3%) was also observed as the first two PCs do not explain half of the variance of the sample. The third PC is characterized by changes in the exterior surface of the marks related to relative changes of the upper and lower halves (constrained *versus* elongated lower half) and shape differences in the interior area of the marks (very expanded interior areas *versus* elongated interior areas located in the lower half of the mark). The most lengthened shape extreme described by PC3 (11.8%) is less pronounced when wolves are excluded, and the exterior and interior surfaces assemble curved shapes.

Nevertheless, the shape changes expressed in the PCA plot (Fig 4A) only describe a small part of the shape variance of the sample, since the first two PCs explain only 38.4% of the total variance. On the contrary, the variance expressed in the scatter-plot in form space is mostly explained by PC1 (87.6%/86.2%) alone, being PC2 (3.3%/3.1%) of far less significance. These results might indicate that centroid size is an important factor affecting the variance of the sample, which is not surprising when carnivore pits are included in the sample because carnivore groups could already be identified based on pits measurements (e.g. [50–52]).

In form space (Fig 5) the variables that appear to mostly explain the variance in shape space, are comprised in the second PC. PC1 in form space is related to changes in the overall form of the pits with rhomboidal and deeper marks at the positive limit of the x-axis and circular and flatter marks at the negative limit of the x-axis. Relative changes in size of the inner and outer surface of the marks are also expressed by PC1 in form space. PC2 and PC3 in form space are determined by the same variables that characterize PC1 and PC2 in shape space, respectively. In form space, changes in depth are more prominent along PC2.

All marks in form space are mostly explained by PC1 (Fig 5). Carnivore pits and percussion marks show similar scattering ranges along PC1. Only when plotting the form PCA results using individual colors for each carnivore we acknowledge some differences regarding hyenas and jaguars, whose pits are less dispersed along the x-axis and show more vertical variability. Unlike in the case of percussion marks, generally carnivore pits tend to gather in the negative range of the y-axis, being related to more oval/circular forms.

Though the PCA graphs (Figs 4 and 5) provide a first impression of the sample distribution according to its morphological variance, further tests have to be conducted in order to assess the entirety of the variance expressed by the sample and obtain numerical results and significance values. The MANOVAs performed on the PC scores to assess the differences among group means show significant results in shape space when only tooth pits as a unique group is compared with percussion marks (F = 6.345, p < 0.0001) and when the pits generated by the individual carnivore species are compared with percussion marks (Table 3). The Pairwise MANOVA indicate that the mean value of the percussion mark sample is significantly different from the mean values for hyenas, jaguars, lions and wolves (Table 3). Shape differences among carnivore groups are also significant except for the pair jaguar-wolf that, however, approaches the significance level (Table 3).

**Table 3:** MANOVA Pairwise p values for mean group comparison in shape and form space.

	PM		Hyena		Jaguar		Lion		Wolf	
	Shape	Form	Shape	Form	Shape	Form	Shape	Form	Shape	Form
**PM**			0.04	0.009	0.008	0.01	<0.0001	<0.0001	<0.001	0.003
**Hyena**	0.04	0.009			0.047	0.23	0.002	0.02	0.02	0.003
**Jaguar**	0.008	0.01	0.047	0.23			0.036	0.23	0.09	0.04
**Lion**	<0.0001	<0.0001	0.002	0.02	0.0036	0.23			0.003	0.002
**Wolf**	<0.001	0.003	0.02	0.003	0.09	0.04	0.003	0.002		

When the data are scaled to centroid size and the MANOVA is performed on the PC scores obtained in form space, percussion and tooth pits can be confidently separated (F = 7.429, p<0.0001). Likewise, the independent carnivore pits can be significantly separated from the percussion marks when form is considered (Table 3). Non-significant values can only be observed between certain carnivore groups: lion-jaguar, hyena-jaguar.

Previous analyses [31] demonstrated that the inclusion of the interior area of carnivore pits caused different results than when only comparing the external morphology of the mark. In this work, differences in the interior area in relation to the exterior area of the pits were assessed taking into account shape variance as well as changes related to the relative sizes, positions and orientations of the data. Here we expand this analysis, including several PLS tests on percussion marks. The PLS results (Table 4) show the existence of an overall strong (r = 0.688) and significant correlation (p < 0.001) between the structure of the inner and outer surface of the percussion marks. Surprisingly, PLS results on only the shape or the size variable of the inner and outer areas, show higher correlation rates than the within-configuration PLS that addresses not only these two factors, but also the relative position/orientation of the outer and inner structures. The covariation between the inner and the outer areas seem to be slightly more strongly determined by the size (r = 0.897, p < 0.0001) than by the shape (r = 0.734, p < 0.0001). Thus, the relative position of the interior and exterior areas might not be as associated as size and shape characteristics among percussion marks.

**Table 4:** PLS results obtained for the comparisons of the internal and external features of the percussion marks (PM) and tooth marks (TM).

Comparison	Sample	RV coefficient	p-value
Interior vs. exterior structure of PM*	PM sample	0.688	<0.001
Interior vs. exterior shape of PM	PM sample	0.734	<0.0001
Interior vs. exterior size of PM	PM sample	0.897	<0.0001
Interior vs. exterior structure of TM*	TM sample**	0.435	<0.001
Interior vs. exterior shape of TM	TM sample**	0.324	<0.0001
Interior vs. exterior size of TM	TM sample**	0.921	<0.0001
Interior vs. exterior structure of TM*	Lion	0.685	0.004
Interior vs. exterior structure of TM*	Hyena	0.557	0.152
Interior vs. exterior structure of TM*	Jaguar	0.701	0.024
Interior vs. exterior structure of TM*	Wolf	0.642	0.024

*Tests assessing the structure of the pits include differences in shape, size and relative positions of the interior and exterior areas.

** Pits sample used in [31], which included crocodile tooth marks.

The correlation of the interior and exterior areas of the percussion marks is similar to those calculated for pits generated by lions, jaguars and wolves, with overall strong and significant correlations between the mark structures (Table 4). Contrary to what happened when exploring the whole carnivore pit sample that shape appeared as a more varying factor with a weak correlation value, among percussion marks the outer and inner shape is clearly correlated, varying in association. Such differences highlight greater inner and outer shape variability among pits, than among percussion marks and might indicate the presence of a morphological pattern among percussion marks when observing the inner and outer morphologies and their relationship.

**Fig 6:**
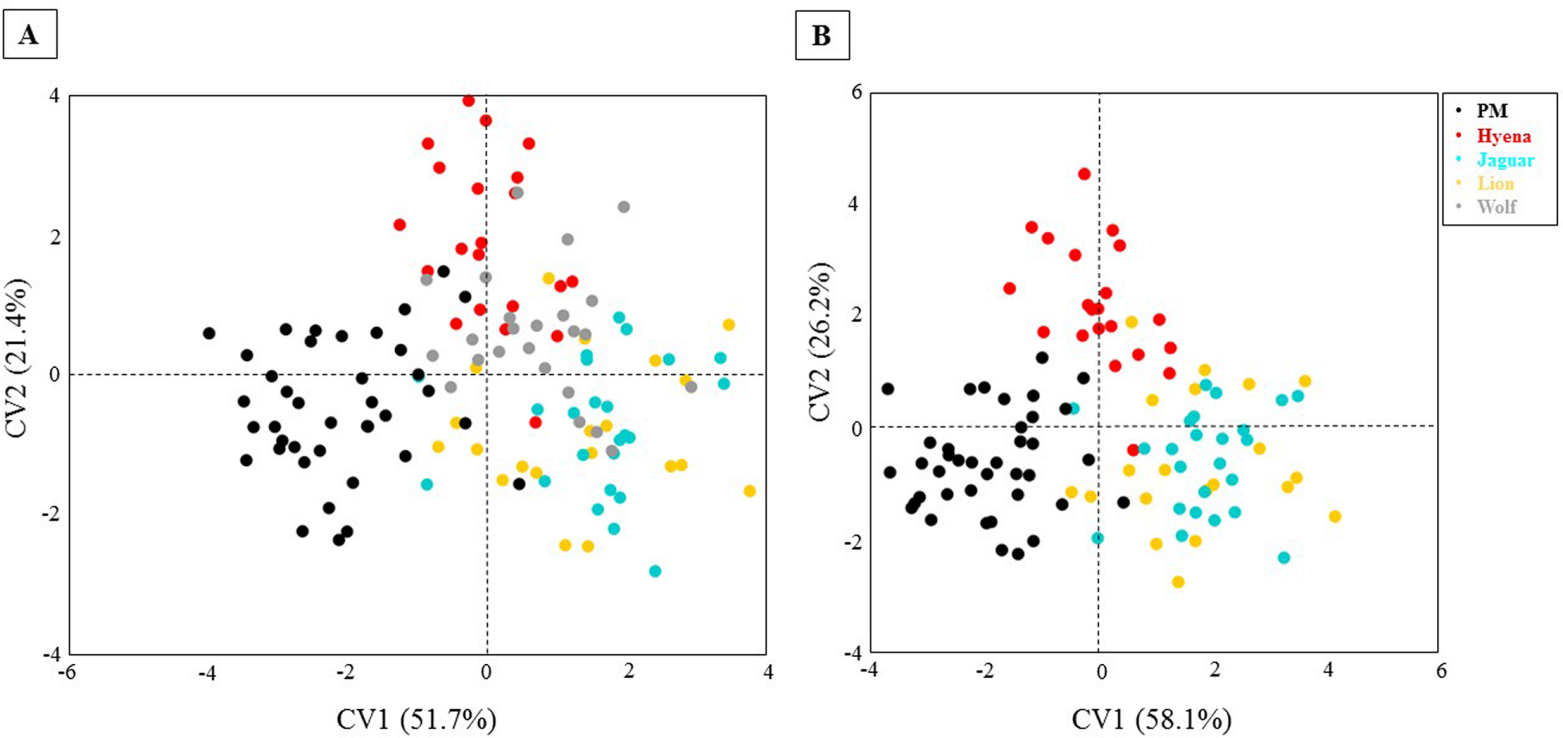
CVA scatter-plots. A) including the whole carnivore pit and percussion sample, B) exluding wolves.

The CVA conducted for the percussion marks and the entire carnivore sample divided by carnivore species support these results. The CVA is explained by four canonical variates (CVs) that account for the variation among the five groups a priori established. Between-group variation is scaled by the inverse of the within-group variation. The CVA graph (Fig 6A) shows the dispersion of the pits per carnivore and the percussion marks according to the first two CVs that explain 73.2% of the differences. The plot can be divided in two halves: one is occupied by the percussion marks group, and along the other half carnivore pits are represented with hyenas and wolves forming two more or less independent groups, and jaguars and lions clearly overlapping in the lowest right corner of the graph. Despite some graphical overlapping, all the Mahalanobis distances calculated highlight the existence of significant differences among all pairs of groups, while the Procrustes distances calculated between jaguars-hyenas and jaguars-wolves are not significant, though very close to the 5% limit of significance (Table 5).

**Table 5:** CVA p values from permutation tests obtained for Mahalanobis and Procrustes distances.

		Hyena		Jaguar		Lion		Wolf
		A*	B**	A*	B**	A*	B**	A*
Mahalanobis	**Hyena**			<0.0001	<0.0001	<0.0001	<0.0001	<0.0001
Procrustes				0.05	0.05	0.003	0.002	0.02
Mahalanobis	**Jaguar**	<0.0001	<0.0001			<0.001	<0.001	<0.0001
Procrustes		0.05	0.05			0.03	0.04	0.08
Mahalanobis	**Lion**	<0.0001	<0.0001	<0.001	<0.001			<0.0001
Procrustes		0.003	0.002	0.03	0.04			0.003
Mahalanobis	**Wolf**	<0.0001		<0.0001		<0.0001		
Procrustes		0.02		0.08		0.003		
Mahalanobis	**PM**	<0.0001	<0.0001	<0.0001	<0.0001	<0.0001	<0.0001	<0.0001
Procrustes		0.03	0.03	0.008	0.007	<0.0001	<0.0001	0.0008

*A: analysis using the whole sample, including pits and percussion marks.

**B: analysis excluding wolf pits.

Except for the comparison jaguar-wolf, all the Mahalanobis and Procrustes distances calculated for all the pairs of groups stress the existence of significant differences between groups (Table 5). In fact, the pair jaguar-wolf shows significant p values for the Mahalanobis distance, falling only slightly over the 5% limit in the calculation of the Procrustes distance (p = 0.08). Differences in Mahalanobis and Procrustes distances among groups can indicate anisotropy of the variation within groups [49]. When wolves are excluded from the analysis, all groups can be clearly separated, especially percussion marks from the rest of the carnivore pits (Fig 6B). The distances calculated between the percussion marks and any other pit groups are always the largest and show the lowest p values, highlighting the statistical significance of the group separation.

A pair comparison between percussion marks and the whole sample of pits used in this study was performed using a jackknife cross-validated LDA and extracting a confusion matrix explaining the misclassification rates between groups. The LDA was performed to assess minimal variance within mark groups and maximal variance between percussion marks and pits. The confusion matrix indicates low confusion rates, with always more than 70% of the marks correctly classified. 71.8% (N = 28) of the percussion marks are correctly classified, while 78.7% (N = 70) of the tooth marks are attributed to the right group. The higher success rates obtained for the pits could indicate that results might improve as sample size increases, since the number of total pits (89) is quite larger than the number of percussion marks used in this study (39).

## Discussion and conclusions

The experimental analysis conducted here to differentiate percussion marks from tooth pits has provided promising results. Both types of marks could successfully be distinguished using three-dimensional reconstruction techniques and multivariate statistical analyses based on the geometric morphometrics principle.

To date, some authors [7–9, 13] had identified a list of morphological features that allow the differentiation of percussion marks and pits by means of macroscopic analyses. However, these authors admit that sometimes tooth pits and percussion marks could not be clearly distinguished, especially when percussion marks were produced with unmodified hammerstones. Unmodified hammerstones, regardless of the raw material, have smooth edges that may not leave microstriations in the interior or generate flat bottoms, being easily mistaken with tooth pits [13]. In fact, several experimental studies have demonstrated that percussion marks produced with modified hammersones can be clearly distinguished from pits, whereas 30% of the percussion marks generated using unmodified hammerstones cannot be separated from tooth pits [21, 22].

Although 70% of percussion marks produced with unmodified hammerstones can be correctly classified, still a high frequency of marks is subject to equifinality. Such morphological overlapping can lead to confusion in the interpretation of archaeological sites, especially in those sites where anthropogenic modification is minor or when percussion marks are inconspicuous and subject to debate [53–57]. It is therefore important to rely on techniques that provide the most accurate identification of marks.

Cut and percussion marks are the most common anthropogenic modifications that can be identified among fossil assemblages. The correct identification of these marks and their frequency in the fossil record are key for a precise archaeological interpretation regarding the origins of human behavior. An inaccurate identification of percussion and cut marks can lead to the false conclusion that humans were modifying agents in contexts where there is no such activity. Similarly, the inability to identify cut and percussion marks could depict humans as scavengers where there is actually an early anthropogenic access to meat resources [17]. This is especially important in very old contexts where the access to meat resources is subject to debate. Some works [53, 54, 58] have presented 3 Ma old fossil bones bearing cut and percussion marks, generating a wide discussion [55–57]. This debate is of utmost relevance because the presence of such marks in these chronologies suggests the existence of very old lithic industries and thus the possibility of australopiths manufacturing tools and processing carcasses. To date the oldest stone tools discovered date 2.6 Ma [59], except for the stone tools identified in Lomekwi 3, West Turkana and dated >3 Ma [60, 61]. Nevertheless, the Lomekwi lithic industry has been called into question from a geological perspective [61].

Classically, cut marks can only be mistaken for trampling marks or tooth scores produced by carnivores. However, many studies have demonstrated that it is possible to distinguish trampling and cut marks based on their morphological characteristics [62–66], the same way scores and cut marks can be identified paying attention to certain features [3, 5, 13]. Similarly, percussion marks and tooth pits show some differences that allow their distinction [7–9, 13, 21], though confusion rates between percussion marks and pits are still important.

The experimental study presented here offers a preliminary solution to this equifinality problem by means of three-dimensional virtual reconstruction of percussion marks and tooth pits, followed by a geometric morphometric analysis. Using this methodology, we could differentiate both types of marks. Although it is true that some marks still show some overlapping (Figs 4 and 5), the global confusion rate is very small, since only inconspicuous percussion marks (produced with unmodified hammerstones and not associated with microstriations) were included in the study. That means that around 70–80% of the percussion marks open to discussion (30% of the global as suggested by [21] and [22]), could be correctly identified according to the results we obtained by means of CVA and LDA where differences among mark groups were significant (p values < 0.05). Altogether, our results reduce the total percentage of misinterpreted percussion marks to 5%.

In addition, our analysis does not only isolate percussion marks from tooth pits, but is also capable of identifying the carnivore involved—hyenas, jaguars, lions and wolves (Fig 6) based on the pits they generate. Thus, our methodology could be applied to different Plio-Pleistocene contexts in Africa, Europe or America. A previous study [31] has already demonstrated the applicability of three-dimensional morphological analyses to carnivore experimental samples and the archeological or paleoanthropological record. The study [31] was able to distinguish between crocodiles, lions, jaguars, wolves and hyenas based on their pit morphology and discussed the ascription of several carnivore pits found on two Olduvai hominins (OH8 and OH35). In the present study, we increased our sample, obtaining more reliable results. However, it is necessary to add more carnivore samples, since certain pits resemble percussion marks more than others. Therefore, it would be interesting to assess similarities among a wider range of tooth pits and compare them also with percussion marks produced with unmodified hammerstones.

Some studies have suggested that virtual reconstruction and geometric morphometric taphonomic analyses are only accurate enough when SEM or other microscopy technology is used [66], but a recent study [29] has shown that micro-photogrammetric techniques offer similar results to those obtained using microscopes. The development of new methodologies applied to the study of taphonomic processes, initiated by [67–69], and continued by [27–29] with applications to the study of cut marks, and tooth marks [31, 32, 70] opens a range of possibilities in the analysis of bone surface modifications, providing precise evidence for the identification of marks. Although our results are still preliminary, our study demonstrates the great potential of the technique when applied to taphonomy.

The further application of these techniques and their future development will certainly improve our diagnostic capability, reducing subjectivity in the archaeological research. In that way, it would be possible to end debate on certain taphonomic processes [53, 54, 58] vs. [56, 61] whose misinterpretation can cause severe damage to the understanding of the early human behavior. Examples of such consequences are widely known: Piltdown hoax or the condemnation of Sanz de Santuola and the Altamira paintings to ostracism.

## Supporting information

S1 Appendix Unmodified hammerstones test. (XLSX)

S2 Appendix Reliability test. (XLSX)

S3 Appendix Percussion and carnivore pits sample. (XLSX)
